# Where and How Is Value Created in Health Systems? Exploring the Role of General Elections

**DOI:** 10.1016/j.jacadv.2024.101369

**Published:** 2024-11-01

**Authors:** Alexander W. Carter, Ankur Kalra

**Affiliations:** a Department of Health Policy, London School of Economics and Political Science, London, United Kingdom; b Franciscan Health, Lafayette, Indiana, USA; c Krannert Cardiovascular Research Center, Indiana University School of Medicine, Indianapolis, Indiana, USA

**Keywords:** general elections, health systems, value, value-based health care, value-based health system

The concept of value-based health systems (VBHS) has recently emerged^1^ as an extension of value-based health care (VBHC). In this politically transformative year, this systems approach to value merits further examination—particularly in terms of what “value” encompasses and how it is created within health systems.^a^ The implications of using this approach for assessing new health technologies and services are explored.

## What is VBHS and why discuss it?

VBHS expands the examination of value beyond traditional methods such as cost-effectiveness analysis (which is integral to Health Technology Assessment [HTA])^b^. VBHS introduces considerations from the political economy, thereby adopting a less technocratic approach to value assessment, which economists have been encouraged to explore.^2^ As such, the forces intersecting with HTA to create value in health systems should be explicitly identified and examined based on their impact. Essentially, HTA is influenced by variables that determine value in different settings and times, yet we lack formal, transparent mechanisms to incorporate these influences into decision-making processes. This misaligns interests across actors and stakeholders, all of whom have a partial and specific role in creating value, thereby creating inefficiencies. We propose a thought experiment: what if the VBHC approach was used to assess every new health intervention throughout its lifecycle? This would involve examining value across entirely new domains beyond those established in HTA, which typically determine costs and benefits at the end stage of clinical investigation.

To reimagine this, VBHS must also be embedded and understood as an approach. The system perspective VBHS encapsulates views of health systems as vehicles for improving societal wellbeing through several causal pathways, although these mechanisms may not be immediately obvious. VBHS represents a significant advancement in identifying these pathways because the conceptual space is deliberately expansive, responding to calls for system-centric approaches to achieve innovation and value in the life sciences.^3^ Elections are a significant component of VBHS because they shape markets, decision-making processes, and outcomes by determining politics and the holders of decision-making power. The broader impact of elections on nonhealth indicators, such as societal wellbeing, is difficult to quantify yet relies on several outcome indicators that are crucial to how we assess value. The extent to which elections are democratic is associated with better health outcomes, as demonstrated by Templin et al^4^ using access as an outcome; this underscores our argument that existing approaches like HTA and VBHS do not adequately consider how politics and power shape the potential for a new intervention to achieve value. Therefore, we lack relevant estimation of the immediate or future value that will be realized given the political economy.

## Applying VBHS to decision-making and politics

Societal wellbeing is a challenging outcome to measure, which is why the quality-adjusted life year (QALY) is such an asset in both VBHC and VBHS, despite its limitations.^5^ For our thought experiment, we focus on the process of value creation that VBHS offers, adopting the same expectations that HTA sets: if we use a transparent process to identify costs, benefits, and uncertainties associated with a health technology, then added value will result. Decision-making is a process of assessing value in the context of values (both of which are shaped by politics and power), leading to HTA recommendations; however, the extension of HTA/value assessment to incorporate VBHS is essential to address what economists refer to as “omitted variable bias” in the form of political economy.

Progress in the use of HTA in decision-making as a means of achieving VBHC is testament to its impact. However, these methods are not magic bullets; eg, the use of QALYs has been questioned based on: 1) whose health and wellbeing they capture; and 2) technical shortcomings that explicitly penalize certain groups. These issues have been addressed by the adoption of QALY modifiers by the National Institute for Health and Care Excellence, although this does not fully resolve how value and values are identified and examined in the decision-making process.

## Axioms of value creation in health systems

The choice of government is a major driver of the decision-making processes adopted by health systems because value and values are shaped by them. This higher-order event shapes all activities associated with VBHC and VBHS. As such, events that have more influence on value creation should be part of value assessment methods.

The urgency to start this effort is evident during a period when elections are reshaping the political economy, with around 50% of the global population voting in elections in 2024. The reason to examine value creation using VBHS is to augment established value assessment methods. This approach is distinct from developments in value assessment that are related but separate from our viewpoint. For example, extending economic evaluation and value assessment methods beyond measuring quality and quantity of life (ie, QALYs) has been argued in the context of VBHC in recent years.^6^ From this, new measures of benefit, such as “real option value” and the “value of hope” provided by treatments, have been proposed. More recently, weights have been attached to these newly proposed measures.^7^ The extent to which these concepts and indicators can capture societal wellbeing is subject to ongoing debate; the most important signal that this provides is that value assessment is evolving, and the system-centric perspective has been invited in.

In our view, it is timely for VBHS to be adopted into practice. The effect of election politics and the power held by actors has a real effect on decision-making and decisions that need to be estimated for any new intervention. At its simplest, we should ask the question: What effects are elections going to have on the perceived value of the intervention? At a minimum, we should expect higher levels of institutional uncertainty that translate directly into decision-making uncertainty, leading us to a revised probability of success (and potential solutions). We could repeat the exercise at different stages of development, including postmarket approval, and routinely update probabilities based on actors’ views of the system. By fostering better alignment between actors’ during the decision-making process, more value will be created because values will be brokered transparently, resulting in better decisions for the system, faster.

These system-centric issues necessitate multidisciplinary and interdisciplinary knowledge to address them. Health economists will need to collaborate with political scientists, sociologists, anthropologists, implementation scientists, etc., under the VBHS framework. This approach should also incorporate the needs of policymakers, who require tools to enhance their practice.^8^

To estimate the effect of elections is to recognize that the system shapes the added value achieved by a technology in various ways. Efforts to reduce the time it takes to implement technologies into practice offer a blueprint for economists.^9^ Delayed implementation changes the added value of a technology. Over a 10-year time horizon for which costs and benefits of technology could be assessed, delays due to higher-order political issues could mean that zero added value is created, regardless of modeled net benefits from HTA. Incorporating this uncertainty, expressed as a probability, into assessments could lead to a different decision, an alternative intervention that citizens can access sooner. Alternatively, it could clearly signal the real source(s) of inefficiency, which is a prerequisite to resolving them.

## Conclusions

Global elections are reshaping decision-making processes, and these extend to health systems. VBHS is a concept that complements developments in the fields of VBHC and HTA because it is self-evident that value creation results from an interplay of factors such as the politics involved and power forged in elections, which could be formalized in value assessment methods. A multidisciplinary and interdisciplinary effort is needed to support innovation and to increase added value from interventions.

## Funding support and author disclosures

The authors have reported that they have no relationships relevant to the contents of this paper to disclose.

## Footnotes

a In this manuscript, we use “value” as a synonym for societal wellbeing or utility, thereby aligning ourselves to welfarist principles, which apply to all governmental systems as it is a theoretical framework used to examine (the attainment of) welfare. As such, its measurement involves several outcome indicators, which we cannot list here in part because these are context-specific.

## Footnotes

b HTA and VBHC are considered equivalent in this article—HTA is the accepted methodological approach to VBHC. We discuss new measures of benefit that are extensions of QALYs that are used in HTA; these extensions are part of broader developments in VBHC.
